# The role of weather conditions on running performance in the Boston Marathon from 1972 to 2018

**DOI:** 10.1371/journal.pone.0212797

**Published:** 2019-03-08

**Authors:** Beat Knechtle, Stefania Di Gangi, Christoph Alexander Rüst, Elias Villiger, Thomas Rosemann, Pantelis Theo Nikolaidis

**Affiliations:** 1 Medbase St. Gallen Am Vadianplatz, St. Gallen, Switzerland; 2 Institute of Primary Care, University of Zurich, Zurich, Switzerland; 3 Exercise Physiology Laboratory, Nikaia, Greece; University of Bourgogne France Comté, FRANCE

## Abstract

This study examined the relationship of weather conditions, together with sex and country of origin, with running performance in the Boston Marathon from 1972 to 2018. A total of 580,990 observations from 382,209 different finishers were analyzed using Generalized Additive Mixed Models. Different groups and subgroups were considered such as all runners, near elite 101:200 finishers, near elite 21:100, annual top ten finishers and annual winners. Weather conditions, over the hours of the event, were average air temperature (°C), total precipitations (mm), wet-bulb globe temperature (WBGT) (°C), wind speed (km/h), wind direction (head wind, side wind, tail wind) and barometric pressure (hPa). These effects were examined in a multi-variable model, together with: sex, country of origin, calendar year, an interaction term country:sex and a spline smooth term in function of calendar year and sex. The average temperature, when increasing by 1°C, was related to worsened performance (by 00:01:47 h:min:sec for all finishers and by 00:00:20 h:min:sec for annual winners). Also, the pressure and wet-bulb globe temperature, when increasing, were related to worsened performances. Tail wind improved performances of all groups. Increasing precipitation was significantly (*p*<0.05) related to worsened performances in all groups except annual winners. Increasing wind speed was also related to worsened performances in all finishers and near elite groups. Kenyans and Ethiopians were the fastest nationalities. The sex differences (men faster than women in all groups) were the largest in near elite groups. Our findings contributed to the knowledge of the performance in Boston Marathon across calendar years, considering as main effects weather conditions, country of origin and sex.

## Introduction

To date, the influence of different environmental conditions such as ambient air temperature wind, precipitations, barometric pressure, humidity, dew point, cloud cover, solar irradiation and atmospheric pollutants have been investigated in marathon running performance [1–7]. It is well-known that environmental conditions have an influence on marathon running performance [8–10] where unfavorable weather conditions such as high ambient temperatures impair performance [4] in both slow [1,5] and fast marathoners [2]. Moreover, performance is more negatively affected by environmental conditions for slow runners [3]. The influence of environmental conditions such as air temperature has been investigated in different marathon races for different performance levels such as top three runners, top ten runners, elite to sub-elite runners or all finishers [1,2,7,11]. However, influences on environmental conditions have been mainly investigated only for limited time periods [1–3], whereas no study has covered a period of longer than 36 years.

The Boston Marathon has the longest tradition in marathon running (it started in 1897 and the first woman participated in 1972) and several studies investigated the influence of environmental conditions on race performance [1,3,11]. However, the longest investigated period for this race was 36 years and concerned elite and sub-elite performances instead of all finishers [3]. Therefore, an analysis since the first women entered the race in 1972 and with all female and male finishers including the aspect of nationality is missing.

The aim of the present study was to investigate the role of weather conditions, together with sex and country, on female and male performance in the Boston Marathon from 1972 to 2018 since in 1972 the first women officially participated in a marathon. We considered the effects, over the hours of the event, of average air temperature (°C), total precipitations (mm), wet-bulb globe temperature (WBGT) (°C), wind speed (km/h), wind direction (*i*.*e*. head wind, side wind, tail wind) and barometric pressure (hPa). These effects were analyzed together with sex, country of origin and calendar year. Based upon previous research, we hypothesized that increasing air temperature impaired running performance in both elite and sub-elite runners.

## Material and methods

### Ethical approval

This study was approved by the Institutional Review Board of Kanton St. Gallen, Switzerland, with a waiver of the requirement for informed consent of the participants as the study involved the analysis of publicly available data.

### Data sampling and data analysis

The Boston Marathon is the world’s oldest annual marathon (www.baa.org/races/boston-marathon/boston-marathon-history.aspx). To compete in this race, athletes must meet time standards which correspond to age and sex (www.baa.org/races/boston-marathon/participant-information/qualifying.aspx). Data are freely available from the Boston Athletic Association website (www.baa.org) and Marathon Guide website (www.marathonguide.com). Data involved in this study are race results from 1972 to 2018 for women and men. Available information from the race records were name and surname of the runners, sex and runners’ nationality, year of competition, and race times. We cleaned the dataset correcting for double coding of the same level of categories (*i*.*e*. female abbreviated with both ‘F’ and ‘W’, single nationalities with many country codes) as reported earlier [12,13]. Moreover, we removed runners with missing performance or questionable (unreliable) information on race time. Unfortunately, we did not have the complete list of all runners belonging to push rim wheelchair division, started on 1975 for men and on 1977 for women. We excluded this category eliminating runners with race time shorter than the annual top record. To identify observations from a single runner, we defined an id variable with name, surname, sex, nationality and period of competition, supposing that a single runner could participate at most for 25 years. Temperature, speed and direction of wind, seemed to have an influence on race time in the Boston Marathon [1,3,4,7,11]. Therefore, we merged the database with information on the weather conditions during the hours of the event. Hourly weather data were obtained from www.wunderground.com/history/daily/us/ma/boston/KBOS and all units were converted to the metric system. All weather data was stored in S1 Dataset. The duration interval of the race has been assumed to be: 9 a.m.− 16 p.m. for all finishers, 9 a.m. − 13 p.m. for near elite groups and annual top ten finishers and 9 a.m. − 12 p.m. for annual winners. Thus, air temperature (°C), wet-bulb globe temperature (WBGT) (°C) [14], wind speed (km/h) and barometric pressure (hPa) were the average of the corresponding hourly values. Wet-bulb globe temperature was calculated with www.kwangu.com/work/psychrometric.htm using the dry bulb temperature and relative humidity obtained from www.wunderground.com/ and an altitude of 43 m above sea level. The total precipitation (mm) was the sum over the duration of the race of the hourly amount of precipitations. The wind direction was the most frequent determination over the hourly observations which were classified as: head wind (*i*.*e*. wind from E, ENE, and ESE), side wind (*i*.*e*. wind from N, NNE, NW, S, SE, SSW, and SW) and tail wind (*i*.*e*. wind from W, WNW, and WSW) [15] due to the race course (www.baa.org/races/boston-marathon/course-map).

### Statistical analysis

Descriptive statistics were presented as means ± standard deviations for continuous variables and as number N (%) for categorical variables. Performance (*i*.*e*. or race time) was recorded in the format “hours:minutes:seconds”. Average performances, by sex and weather conditions, were reported for the following groups: temperature of 0–7 °C, 8–15 °C and 16–24 °C; wind direction head, side or tail; total precipitations equal 0 and >0 mm; WBGT of 0–6 °C, 7–10 °C, 11–15 °C and 16–20 °C; wind speed of 9–17 km/h, 18–24 km/h and 25–39 km/h; pressure <1015 hPa and ≥1015 hPa. The effects of calendar year on race time, together with the effects of sex, country of origin and weather conditions, were examined through multi-variable statistical models. Results were presented as estimates (standard errors). Different analyses and regression models were performed for the following subgroups: all runners, annual top 101:200, annual top 21:100, annual top ten finishers and annual winners. The calendar year of the race was considered as a discrete value of a continuous variable. The country groups included the 8 most prevalent geographical areas in terms of participation (*i*.*e*. Africa, Kenya-Ethiopia, Asia, Canada, Central-South America, Europe, Oceania, and USA). For annual top finishers (winners) country groups were only 4: Kenya-Ethiopia, Europe, USA and other countries. Weather characteristics such as: temperature, precipitations, WBGT, wind speed and pressure were considered as continuous variables. Wind direction as a categorical variable. The acceptable type I error was set at *p*<0.05. Preliminary data visualization and previous research [12,13] justified the choice of spline regression models for the underlying temporal trend of performance of all groups of finishers. Moreover, random effects on intercept, at runner’s level, accounted for repeated measurements within finishers. Therefore, we performed Generalized Additive Mixed Models specified as follows:

$$\begin{array}{ll} \text{Race}\;\text{Time}(\text{Y})\sim & [\text{Fixed}\;\text{effects}(\text{X})=\text{Sex}+\text{Country}+\text{Weather}\;\text{Conditions}+\\ & \text{Sex}:\text{Country}+\text{S}(\text{YEAR},\text{k}=9,\text{by}=\text{sex})]+\\ & \left[\text{Random}\;\text{effects}\;\text{of}\;\text{intercept}=\text{Runners}\right] \end{array}$$ (1)

where Sex: Country was the interaction term between country groups and sex and S(YEAR, k = 9, by = sex) was a 9-dimension spline, changing over calendar year and with sex. The interaction term was dropped in the models for near elite groups and annual top ten group because it was not significant and removing it from the models improved the fit. For annual top 101:200 analysis, also country effect alone was removed for the same reason. Instead, in annual winners’ model WBGT was dropped. For the most relevant predictors, we reported 3-dimensional perspective plot views or partial or summed effects plots where appropriate. Partial effects were the isolated effects of one particular predictor or interaction. Summed effects were the predicted values for a certain situation or condition, so that all the partial effects that apply to that situation were summed up, including the intercept. All statistical analyses were carried out using statistical software R, R Core Team (2016). R: A language and environment for statistical computing. R Foundation for Statistical Computing, Vienna, Austria, URL https://www.R-project.org/. In particular, we used the following R packages: ggplot2 for preliminary data visualization; gamm4 for multivariable mixed models with random effects on intercept; mgcv for statistical models visualization. The R script, used to manage data and to run the analyses, was provided in S1 File.

## Results

Between 1972 and 2018, a total of 580,990 observations from 382,209 different finishers were recorded in the race results. Therefore we had many observations per runner.

### Summary statistics

In Table 1, for each group of finishers, we reported the summary statistics of the average performance by sex and by weather conditions. Considering average temperature, in most cases, performances were better for men with 8–15 °C and for women with 0–7 °C. For example, for annual top ten, the average fastest time for women was 02:33:34±00:06:59 h:min:sec (temperature below 8 °C) and for men 02:12:38±00:03:46 h:min:sec (temperature 8–15 °C). When considering wind direction, men performed better when there was tail wind (*i*.*e*. 02:09:50±00:03:35 h:min:sec for men winners). Instead, women performed better when there was head wind in most cases. Performances were also better, most frequently, with absence of precipitations for women and for men, on the contrary, with presence of precipitations. When considering wet-bulb globe temperature, when the level was 0–6 °C, both men and women performed better in all cases except all male finishers. In most cases, when wind speed was 18–24 km/h, men and women performed better. Both sexes performed better, in most cases for women and in all cases for men, when pressure <1015 hPa. In S1 Table the frequency distribution of time-performance groups, of all finishers, by sex and weather conditions, was reported.

**Table 1:** Race time (Mean and SD), by sex and weather conditions, for all groups of finishers.

All finishers N = 580,990					Top 101:200 N = 8,687			Top 21:100 N = 7,148			Top 10 N = 938			Winners N = 94		
**Temperature (°C)**	**Sex**	**N**	**Mean**	**SD**	**N**	**Mean**	**SD**	**N**	**Mean**	**SD**	**N**	**Mean**	**SD**	**N**	**Mean**	**SD**
0–7	F	43,281	04:11:30	00:44:05	800	03:11:48	00:05:47	640	02:59:18	00:06:23	80	02:33:34	00:06:59	8	02:29:00	00:05:41
0–7	M	85,233	03:44:51	00:47:56	800	02:35:49	00:05:55	640	02:28:10	00:06:22	80	02:13:21	00:03:54	8	02:10:25	00:02:17
8–15	F	120,137	04:02:49	00:36:58	2,486	03:14:10	00:07:00	2,018	03:01:14	00:07:54	278	02:35:56	00:18:36	28	02:28:56	00:10:35
8–15	M	227,503	03:36:25	00:39:46	2,800	02:37:26	00:06:05	2,240	02:28:39	00:06:11	280	02:12:38	00:03:46	28	02:09:36	00:03:09
16–24	F	36,638	04:21:18	00:42:57	802	03:19:21	00:08:20	730	03:09:23	00:12:32	110	02:44:29	00:19:24	11	02:35:22	00:13:26
16–24	M	68,198	03:50:44	00:48:40	999	02:42:12	00:06:04	880	02:33:27	00:06:04	110	02:16:31	00:03:51	11	02:12:31	00:02:04
**Wind direction**	**Sex**	**N**	**Mean**	**SD**	**N**	**Mean**	**SD**	**N**	**Mean**	**SD**	**N**	**Mean**	**SD**	**N**	**Mean**	**SD**
Head wind	F	109,175	04:06:35	00:38:49	1,302	03:12:33	00:06:44	1,130	03:03:03	00:10:55	150	02:35:49	00:10:45	16	02:29:39	00:06:27
Head wind	M	188,902	03:43:59	00:42:52	1,500	02:40:01	00:05:46	1,200	02:31:26	00:06:16	150	02:14:03	00:03:59	16	02:11:08	00:03:22
Side wind	F	64,725	04:12:36	00:43:44	1,886	03:16:08	00:07:54	1,528	03:02:38	00:08:25	208	02:37:15	00:20:21	21	02:30:17	00:12:04
Side wind	M	138,749	03:40:03	00:46:05	2,100	02:37:38	00:06:40	1,680	02:29:14	00:06:31	210	02:13:42	00:04:05	21	02:10:10	00:02:25
Tail wind	F	26,156	04:03:10	00:36:52	900	03:14:54	00:06:52	730	03:01:58	00:09:36	110	02:40:24	00:20:01	10	02:32:04	00:14:16
Tail wind	M	53,283	03:31:56	00:39:10	999	02:36:36	00:06:15	880	02:28:11	00:06:24	110	02:13:06	00:04:22	10	02:09:50	00:03:35
**Precipitations**	**Sex**	**N**	**Mean**	**SD**	**N**	**Mean**	**SD**	**N**	**Mean**	**SD**	**N**	**Mean**	**SD**	**N**	**Mean**	**SD**
0	F	187,393	04:07:34	00:39:22	3,888	03:15:02	00:07:32	3,228	03:02:49	00:09:43	448	02:37:25	00:18:09	45	02:30:16	00:10:59
0	M	358,780	03:41:01	00:43:12	4,399	02:38:21	00:06:23	3,600	02:29:43	00:06:31	450	02:13:27	00:04:00	45	02:10:18	00:02:57
> 0 mm	F	12,663	04:15:39	00:52:30	200	03:08:45	00:02:36	160	02:58:51	00:04:20	20	02:40:08	00:05:57	2	02:34:41	00:07:23
> 0 mm	M	22,154	03:38:22	00:52:05	200	02:34:30	00:06:28	160	02:29:15	00:06:44	20	02:18:40	00:03:59	2	02:13:16	00:03:49
**WBGT (°C)**	**Sex**	**N**	**Mean**	**SD**	**N**	**Mean**	**SD**	**N**	**Mean**	**SD**	**N**	**Mean**	**SD**	**N**	**Mean**	**SD**
0–6	F	82,843	04:03:58	00:39:15	1,986	03:12:05	00:07:45	1,608	02:59:28	00:07:35	210	02:33:21	00:09:02	21	02:28:29	00:06:51
0–6	M	165,228	03:37:48	00:42:59	2,100	02:35:28	00:06:13	1,680	02:27:31	00:06:29	210	02:12:00	00:03:24	21	02:09:15	00:02:23
7–10	F	80,620	04:07:43	00:39:36	1,502	03:16:27	00:05:28	1,280	03:04:45	00:09:59	168	02:38:21	00:21:46	17	02:30:18	00:12:01
7–10	M	148,220	03:40:02	00:42:37	1,700	02:39:17	00:05:08	1,360	02:29:59	00:05:35	170	02:13:59	00:03:14	17	02:10:41	00:02:33
11–15	F	21,361	04:05:48	00:37:07	500	03:18:27	00:08:08	420	03:06:45	00:11:18	80	02:46:56	00:22:44	8	02:35:48	00:16:13
11–15	M	44,369	03:36:57	00:39:06	699	02:42:23	00:06:05	640	02:33:45	00:05:58	80	02:17:09	00:05:14	8	02:12:39	00:04:11
16–20	F	15,232	04:35:39	00:43:54	100	03:22:45	00:02:06	80	03:10:29	00:06:09	10	02:36:36	00:03:49	1	02:31:50	
16–20	M	23,117	04:15:36	00:49:48	100	02:47:14	00:01:57	80	02:37:49	00:03:41	10	02:15:26	00:02:06	1	02:12:40	
**Wind speed (km/h)**	**Sex**	**N**	**Mean**	**SD**	**N**	**Mean**	**SD**	**N**	**Mean**	**SD**	**N**	**Mean**	**SD**	**N**	**Mean**	**SD**
9–17	F	55,457	04:11:57	00:42:33	1,586	03:14:56	00:07:55	1,290	03:01:17	00:08:17	188	02:40:06	00:24:59	24	02:32:08	00:13:42
9–17	M	95,768	03:44:43	00:46:14	1,850	02:38:25	00:06:33	1,520	02:29:46	00:06:35	190	02:13:41	00:04:13	24	02:10:50	00:03:06
18–24	F	75,953	04:03:46	00:37:50	1,702	03:16:23	00:06:34	1,440	03:04:54	00:09:55	180	02:35:08	00:07:56	13	02:27:35	00:03:09
18–24	M	180,831	03:35:33	00:41:24	1,800	02:37:39	00:07:20	1,440	02:29:40	00:07:05	180	02:14:05	00:03:47	13	02:09:50	00:02:15
25–39	F	68,646	04:09:44	00:40:50	800	03:10:45	00:06:58	658	03:00:17	00:10:05	100	02:37:03	00:13:23	10	02:30:09	00:09:25
25–39	M	104,335	03:46:32	00:44:21	949	02:38:44	00:03:44	800	02:29:37	00:05:17	100	02:12:53	00:04:27	10	02:10:14	00:03:42
**Pressure (hPa)**	**Sex**	**N**	**Mean**	**SD**	**N**	**Mean**	**SD**	**N**	**Mean**	**SD**	**N**	**Mean**	**SD**	**N**	**Mean**	**SD**
<1015	F	100,015	04:09:21	00:40:49	2,000	03:15:24	00:06:38	1,610	03:02:21	00:08:42	218	02:36:29	00:19:57	21	02:29:21	00:11:08
<1015	M	186,398	03:40:51	00:44:22	2,149	02:37:41	00:07:27	1,760	02:28:47	00:06:47	220	02:12:48	00:03:27	21	02:09:35	00:02:48
> = 1015	F	100,041	04:06:49	00:39:53	2,088	03:14:04	00:08:10	1,778	03:02:53	00:10:18	250	02:38:27	00:15:41	26	02:31:20	00:10:44
> = 1015	M	194,536	03:40:52	00:43:12	2,450	02:38:37	00:05:21	2,000	02:30:30	00:06:11	250	02:14:26	00:04:31	26	02:11:06	00:03:03

### Statistical analysis

In Table 2, the results of the multivariable generalized additive mixed model, described in the methods section, were shown.

**Table 2:** Statistical models: Estimate (std errors) were reported as h:min:sec. For *p*-value see Note. For smoothing terms, a global t-test was reported. For each categorical predictor, the reference category (ref.) was reported.

	All finishers	Top 101:200	Top 21:100	Top 10	Winners
(Intercept)	00:11:24 ***	02:19:43 ***	01:19:43***	01:18:23 **	01:49:23 **
	(00:06:57)	(00:04:49)	(00:08:28)	(00:25:07)	(00:34:15)
**Country (ref. KEN-ETH)**					
Country = Africa	01:43:37***		00:00:53	00:00:46	
	(00:06:17)		(00:01:17)	(00:01:24)	
Country = Asia	01:45:14***		00:04:54 ***	00:02:20*	
	(00:04:44)		(00:00:52)	(00:01:04)	
Country = Canada	01:27:28***		00:07:55 ***	00:02:32	
	(00:04:40)		(00:00:53)	(00:01:31)	
Country = Central-South America	01:22:04***		00:05:09 ***	00:01:46	
	(00:04:45)		(00:00:55)	(00:01:10)	
Country = Europe	01:40:43***		00:04:30 ***	00:01:04	00:02:57
	(00:04:42)		(00:00:51)	(00:00:50)	(00:01:52)
Country = Oceania	01:28:30***		00:03:59 ***	00:02:16	
	(00:04:57)		(00:01:06)	(00:01:38)	
Country = USA	01:36:43***		00:08:19 ***	00:03:00 ***	00:06:30 **
	(00:04:39)		(00:00:49)	(00:00:45)	(00:01:57)
Country = Other					00:08:03 **
					(00:02:45)
**Sex (ref. F)**					
Sex:M	- 00:07:45	- 00:41:51 ***	- 00:34:51 ***	- 00:24:02***	- 00:17:25 ***
	(00:05:25)	(00:00:11)	(00:00:10)	(00:00:29)	(00:01:24)
**Average temperature (C°)**	00:01:47***	00:00:31 ***	00:00:23***	00:00:05	00:00:20 ***
	(00:00:01)	(00:00:01)	(00:00:02)	(00:00:06)	(00:00:05)
**WBGT C°**	00:00:10***	00:00:30 ***	00:00:31***	00:00:22 **	
	(00:00:02)	(00:00:02)	(00:00:03)	(00:00:09)	
**Precipitations (mm)**	00:00:44***	00:00:10 ***	00:00:10***	00:00:10*	00:00:07
	(00:00:01)	(00:00:01)	(00:00:01)	(00:00:04)	(00:00:06)
**Pressure (hPa)**	00:00:06***	00:00:03***	00:00:05***	00:00:04**	00:00:02
	(00:00:00)	(00:00:00)	(00:00:01)	(00:00:01)	(00:00:02)
**Wind speed (km/h)**	00:00:13***	00:00:07 ***	00:00:06***	- 00:00:01	00:00:00
	(00:00:00)	(00:00:00)	(00:00:01)	(00:00:02)	(00:00:03)
**Wind direction (ref. Tail wind)**					
Wind direction = Head wind	00:11:51***	00:03:06 ***	00:03:34***	00:04:31***	00:05:06***
	(00:00:10)	(00:00:09)	(00:00:15)	(00:00:44)	(00:00:59)
Wind direction = Side wind	00:08:04 ***	00:01:37***	00:01:17***	00:01:57**	00:02:06*
	(00:00:09)	(00:00:08)	(00:00:13)	(00:00:37)	(00:00:49)
**Interaction Country:Sex**					
Africa:M	- 00:24:45***				
	(00:07:11)				
Asia:M	- 00:11:21*				
	(00:05:30)				
Canada:M	- 00:12:22*				
	(00:05:26)				
Central-South America:M	- 00:06:20				
	(00:05:32)				
Europe:M	- 00:14:56**				- 00:00:29
	(00:05:27)				(00:02:54)
Oceania:M	- 00:12:35*				
	(00:05:46)				
USA:M	- 00:11:59*				- 00:06:07*
	(00:05:25)				(00:02:24)
Other:M					- 00:05:32
					(00:03:06)
**Smoothing terms**	p < 0.001, overall	p < 0.001, overall	p < 0.001, overall	p < 0.001, overall	F: p < 0.001 M: p>0.05
s(YEAR)1: F	- 00:07:30	- 02:04:51***	- 00:45:35***	- 01:05:15**	- 00:27:06**
	(00:01:31)	(00:05:33)	(00:02:52)	(00:03:09)	(00:03:45)
s(YEAR)2: F	- 03:44:18***	- 04:29:54***	- 01:39:30	- 02:59:17	- 00:47:06
	(00:16:54)	(00:13:08)	(00:07:00)	(00:08:56)	(00:10:31)
s(YEAR)3: F	00:41:28***	- 01:06:19***	- 00:24:01 ***	- 00:46:21 *	- 00:15:59 **
	(00:02:28)	(00:03:17)	(00:01:52)	(00:02:27)	(00:03:07)
s(YEAR)4: F	01:49:50***	02:49:49***	01:05:20 ***	02:00:19	00:28:46 **
	(00:11:36)	(00:08:47)	(00:04:53)	(00:06:41)	(00:07:35)
s(YEAR)5: F	- 00:35:23***	00:38:03***	00:09:28 ***	00:37:45	00:09:26 ***
	(00:03:05)	(00:02:32)	(00:01:40)	(00:02:47)	(00:03:05)
s(YEAR)6: F	02:19:33***	02:17:24***	00:46:44 ***	01:56:57	00:27:56***
	(00:09:57)	(00:07:37)	(00:04:29)	(00:06:45)	(00:07:11)
s(YEAR)7: F	00:29:11***	- 00:31:49***	- 00:10:27 ***	- 00:36:13	- 00:06:37
	(00:04:06)	(00:01:55)	(00:01:34)	(00:03:06)	(00:03:01)
s(YEAR)8: F	- 06:41:03***	- 07:24:04***	- 02:37:38 ***	- 04:52:05***	- 01:20:38
	(00:32:22)	(00:22:22)	(00:11:40)	(00:14:19)	(00:16:07)
s(YEAR)9: F	- 01:51:11	-03:02:34***	- 00:53:29 ***	- 01:17:45***	- 00:31:38
	(00:08:31)	(00:08:52)	(00:04:44)	(00:06:23)	(00:06:54)
s(YEAR)1: M	- 00:21:29 ***	- 00:21:10***	- 00:13:22 ***	- 00:02:01	- 00:00:15***
	(00:00:40)	(00:00:28)	(00:00:53)	(00:02:18)	(00:00:58)
s(YEAR)2:M	- 00:16:59 ***	- 00:48:29***	- 00:40:09 ***	- 00:20:13 **	- 00:00:50 *
	(00:04:04)	(00:01:29)	(00:02:44)	(00:06:22)	(00:01:32)
s(YEAR)3:M	00:04:24 ***	- 00:08:21***	- 00:05:08 ***	- 00:02:57 *	- 00:00:09
	(00:00:45)	(00:00:19)	(00:00:38)	(00:01:53)	(00:00:26)
s(YEAR)4:M	- 00:29:44 ***	00:30:44***	00:29:07 ***	00:15:41 **	00:00:32
	(00:02:51)	(00:01:08)	(00:02:04)	(00:04:39)	(00:00:53)
s(YEAR)5:M	00:01:42 ***	00:02:15***	00:02:14***	00:01:52	00:00:10*
	(00:00:49)	(00:00:22)	(00:00:44)	(00:01:49)	(00:00:19)
s(YEAR)6:M	00:05:07***	00:19:40***	00:16:07 ***	00:11:15 ***	00:00:31 **
	(00:02:38)	(00:01:08)	(00:02:04)	(00:04:08)	(00:00:46)
s(YEAR)7:M	- 00:07:42***	- 00:03:37***	- 00:02:59 ***	- 00:03:14**	- 00:00:11
	(00:00:59)	(00:00:26)	(00:00:51)	(00:01:39)	(00:00:17)
s(YEAR)8:M	- 00:01:33 ***	- 01:08:51***	- 00:57:38 ***	- 00:33:40**	- 00:02:11**
	(00:07:49)	(00:02:32)	(00:04:32)	(00:09:41)	(00:02:23)
s(YEAR)9:M	- 00:05:05*	-00:17:55***	-00:05:46	00:01:04	- 00:01:30**
	(00:02:11)	(00:01:03)	(00:01:56)	(00:04:06)	(00:01:13)
**N**	**580,924**	**8,687**	**7,133**	**938**	**94**

*p<0.05;

**p<0.01;

***p<0.001.

Smoothing terms = s(YEAR)n:Sex (where n = 1–9 and Sex = F,M). For instance, s(YEAR)1:F was the first smoothing term for Sex = F.

#### Weather conditions

As temperature, WBGT, pressure, precipitations or wind speed increased, performances significantly worsened in most cases. In fact, when average temperature increased by 1 °C, performances were slower with a greater effect for all finishers: 00:01:47 (00:00:01) h:min:sec, *p*<0.001 and a smaller effect for annual winners 00:00:20 (00:00:05) h:min:sec, *p*<0.001. For the annual top ten, a temperature variation effect on performance was not significant. As pressure increased by one hPa, performances significantly worsened with a greater effect for all finishers: 00:00:06 (00:00:00) h:min:sec, *p*<0.001 and a smaller effect for the annual top 101:200 finishers: 00:00:03 (00:00:00) h:min:sec, *p*<0.001. For the annual winners, an effect of pressure variation on performance was not significant. As wind speed increased by 1 km/h, performances significantly worsened with a greater effect for all finishers: 00:00:13 (00:00:00) h:min:sec, *p*<0.001 and a smaller effect for the annual top 21:100: 00:00:06 (00:00:01) h:min:sec, *p*<0.001. For the elite groups (*i*.*e*. the annual top 10 and the annual winners) the effect of wind speed variation on performance was not significant. As precipitations increased by 1 mm, performances significantly worsened, with a greater effect for all finishers: 00:00:44 (00:00:01) h:min:sec, *p*<0.001 and equal effect for the other groups: estimate 00:00:10 h:min:sec. For the annual winners, a precipitations effect was not significant. A wet-bulb globe temperature effect was significant in all groups except winners. Analogously to the other weather effects, as wet-bulb globe temperature increased by 1 °C, performances worsened with a greater effect for near elite groups: 00:00:31 (00:00:03) h:min:sec, *p*<0.001 in top 21:100 group and a smaller effect for all finishers: 00:00:10 (00:00:02) h:min:sec, p<0.001. Performances of all groups were significantly (*p*<0.001, in most cases) slower with head and side wind compared to tail wind. The effects were greater for all finishers: compared to tail wind, head wind slowed down performance by 00:11:51 (00:00:10) h:min:sec, *p*<0.001 and side wind by 00:08:04 (00:00:09) h:min:sec, *p*<0.001. For the near elite finishers, in particular the top 101:200, the effects of wind direction were smaller: head wind slowed down performance by 00:03:06 (00:00:09) h:min:sec, *p*<0.001 and side wind by 00:01:37 (00:00:08) h:min:sec, *p*<0.001.

#### Country, sex and calendar year effects

**Fig 1:**
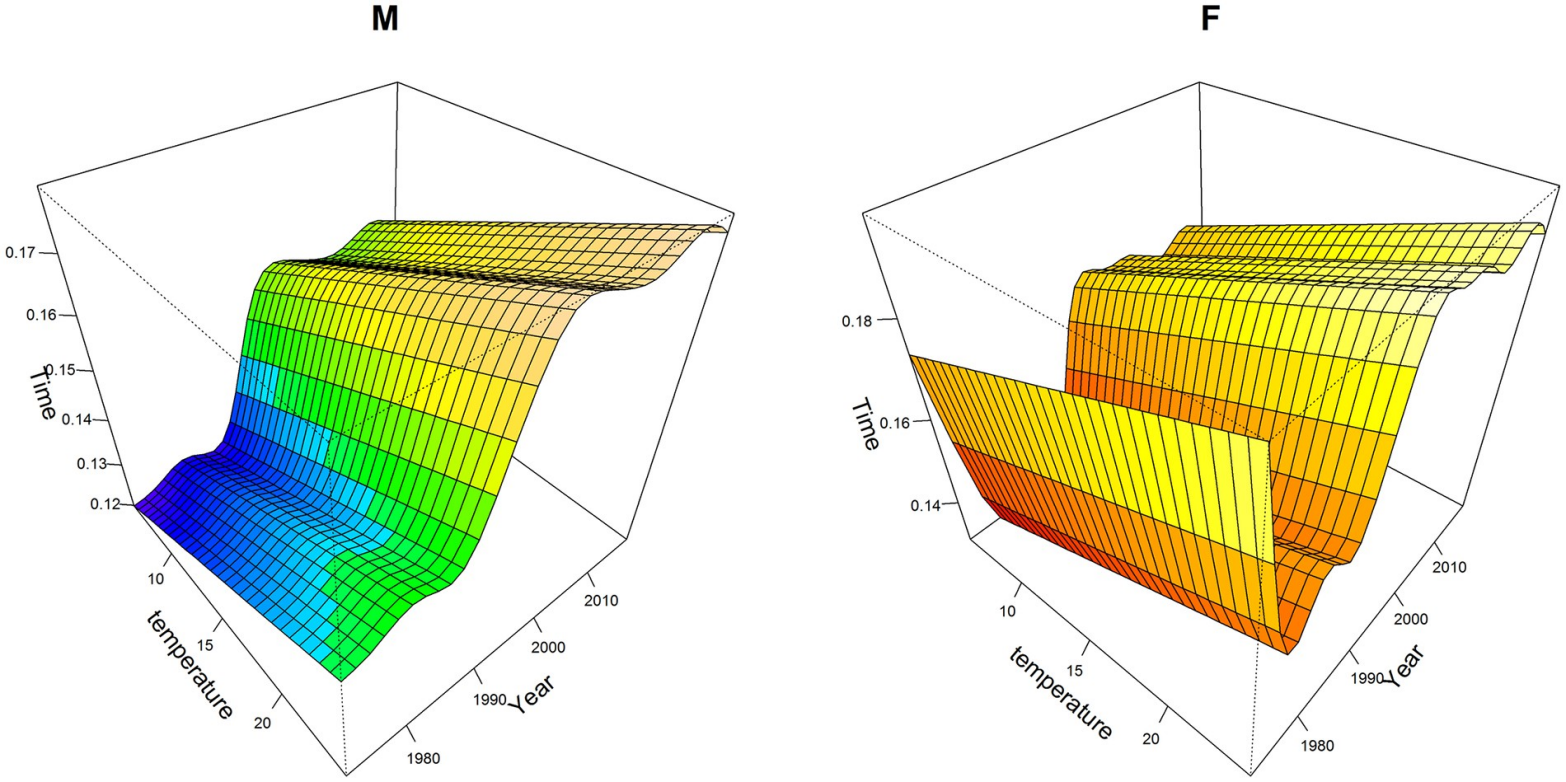
Statistical model of performances of all finishers: Perspective plot views. **Effects of temperature, year and sex**. Time was labelled in fraction of a day, *i*.*e*. 0.125 = 03:00:00 h:min:sec.

**Fig 2:**
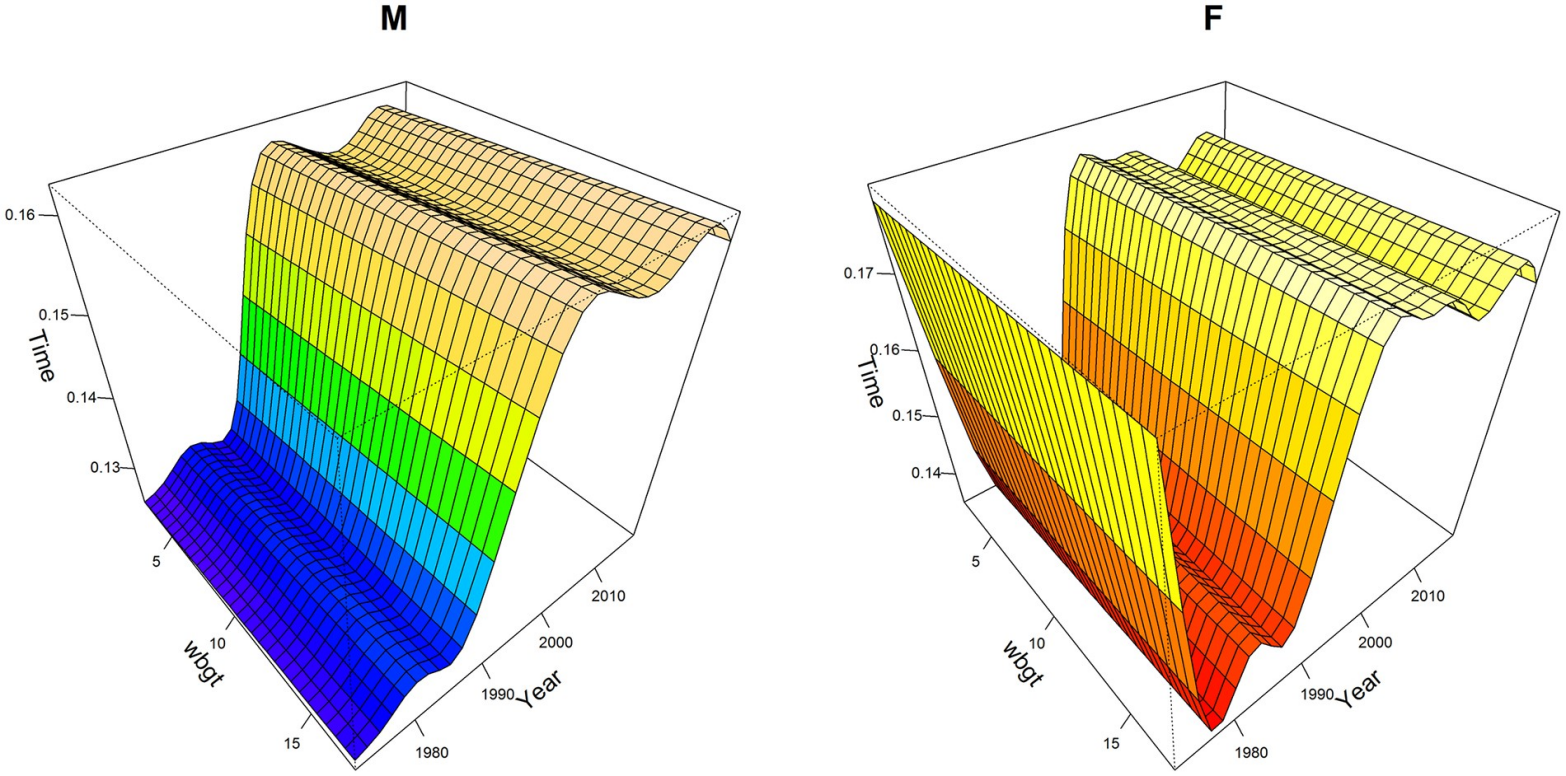
Statistical model of performances of all finishers: Perspective plot views. **Effects of WBGT, year and sex**. Time was labelled in fraction of a day, *i*.*e*. 0.125 = 03:00:00 h:min:sec.

**Fig 3:**
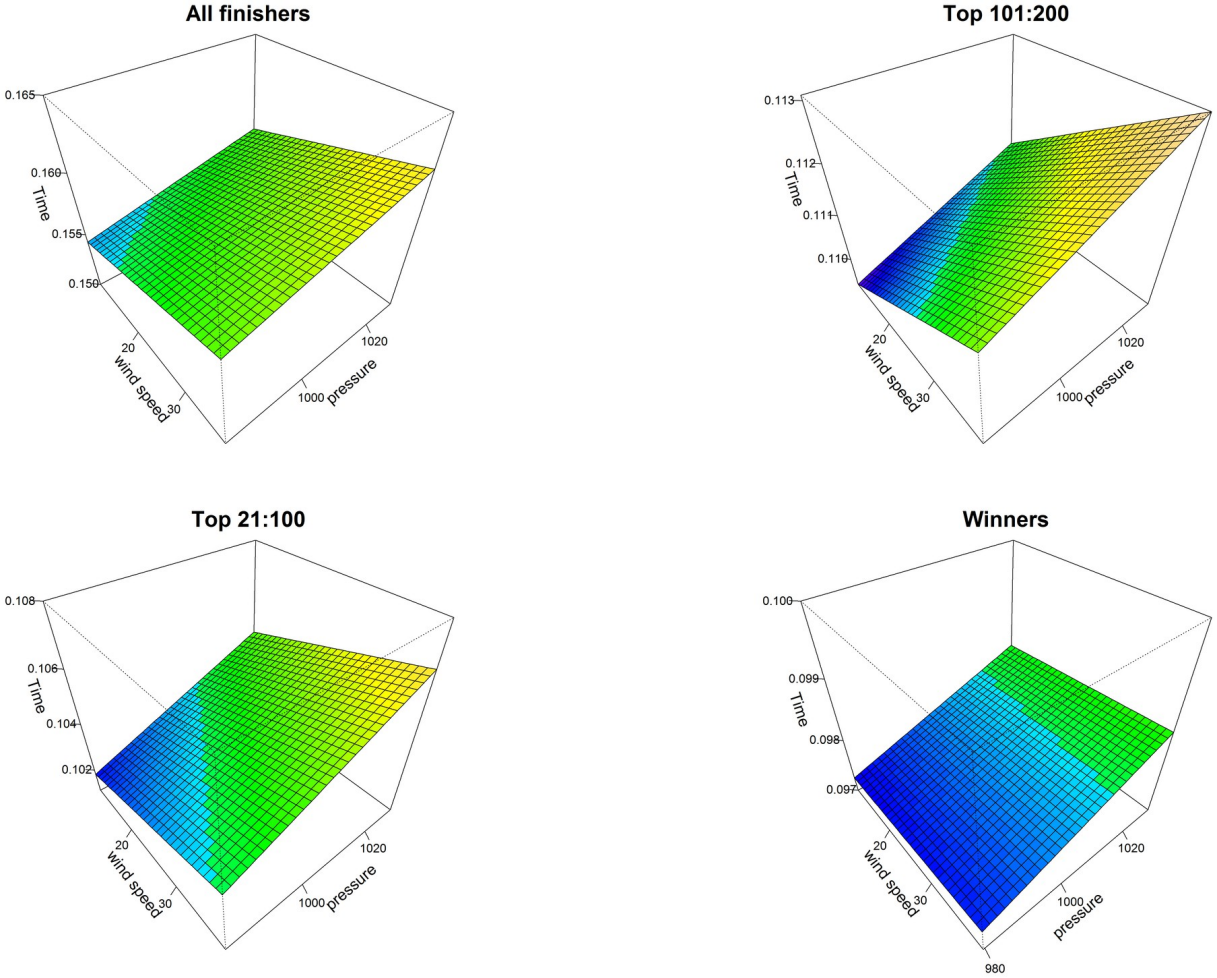
Statistical model of performances of all finishers, near elite groups and winners: Perspective plot views. **Effects of wind speed and pressure**. Time was labelled in fraction of a day, *i*.*e*. 0.125 = 03:00:00 h:min:sec.

**Fig 4:**
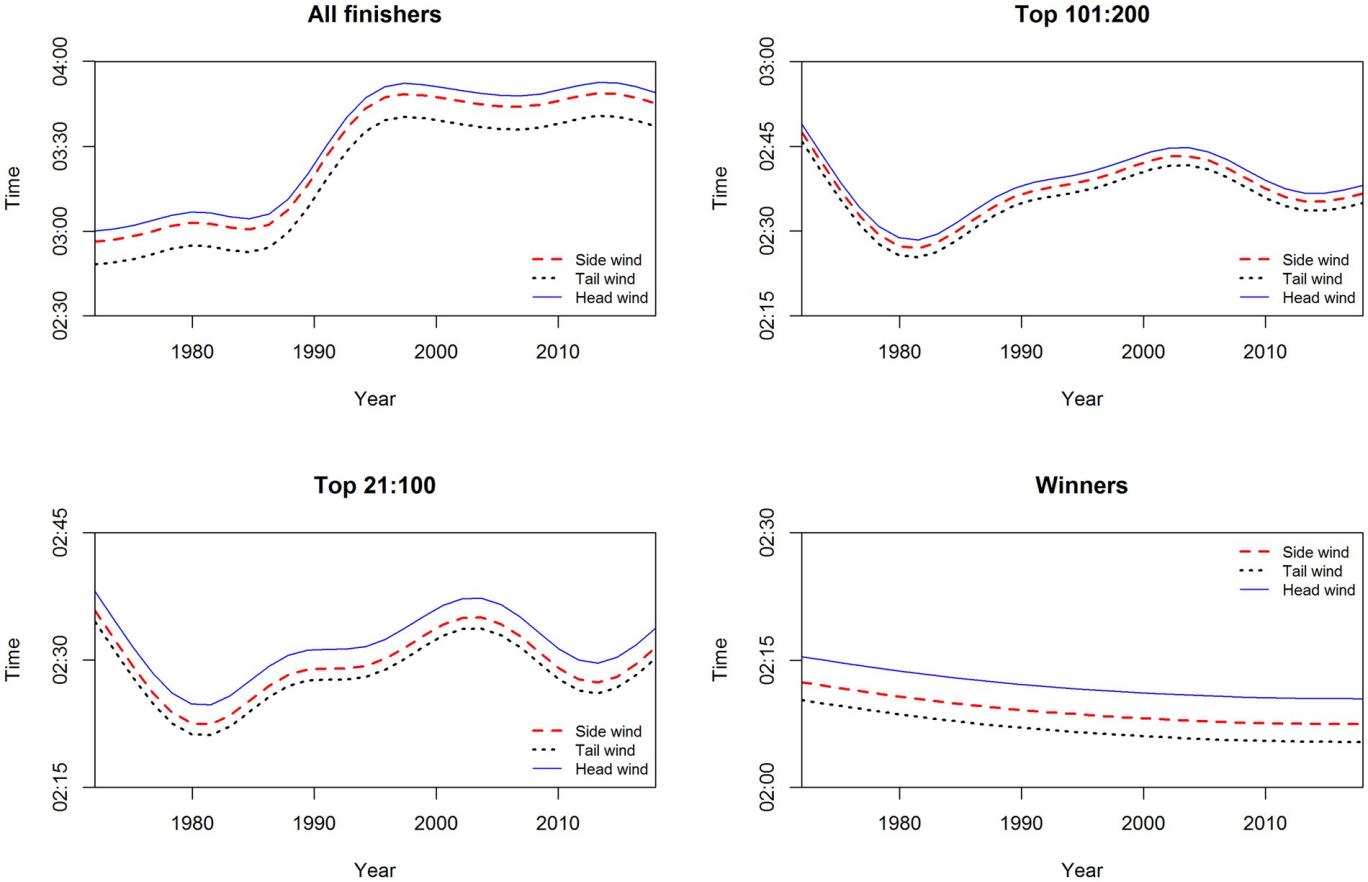
Statistical model of performances of all finishers, near elite groups and winners. Year trend by wind direction.

**Fig 5:**
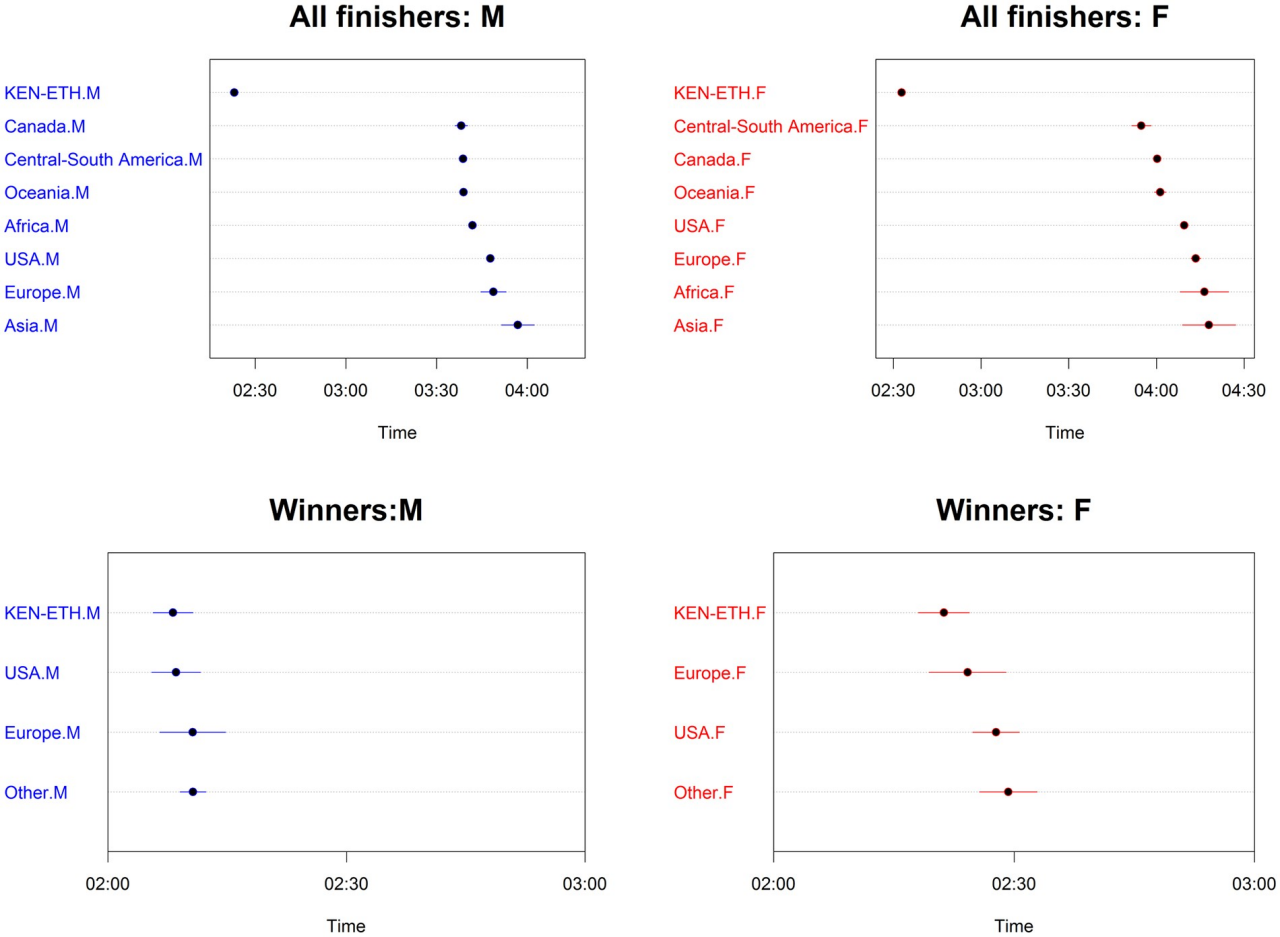
Statistical model of performances of all finishers and annual winners. Effects of country and sex.

Athletes from Kenya and Ethiopia were significantly the fastest compared to every country group. The differences varied between sexes and between groups of finishers. The greater effects were observed for all finishers and the smaller effects for annual top 10 finishers. For women in all finishers group, the differences ranged from a minimum of 01:22:04 (00:04:45) h:min:sec, *p*<0.001, which was the comparison between Kenya—Ethiopia and Central-South America, to a maximum of 01:45:14 (00:04:44) h:min:sec, *p*<0.001, which was the comparison between Kenya-Ethiopia and Asia. In the top ten group, the significant differences ranged from a minimum of 00:02:20 (00:01:04) h:min:sec, *p*<0.01, which was the comparison between Kenya—Ethiopia and Asia, to a maximum of 00:03:00 (00:00:45) h:min:sec, *p*<0.001, which was the comparison between Kenya-Ethiopia and USA. The interaction terms country:sex represented the sex differences for each country group. For instance, the term Africa:M estimated how much greater the effect of being men on race time was for a runner from Africa, compared to a runner from Kenya-Ethiopia. From Table 2, for all finishers, the male effect of Africa runners, compared to the male effect of Kenya-Ethiopia runners, was to improve performance by 00:24:45 (00:07:11) h:min:sec, *p*<0.001. Men were significantly faster than women in all groups, with a greater effect in the near elite groups, in particular the top 101:200 finishers: 00:41:51 (00:00:11) h:min:sec, *p*<0.001 and a smaller effect in annual winners: 00:17:25 (00:01:24) h:min:sec, *p*<0.001. In all finishers group, being men was a significant factor when interacted with country. Moreover, performances changed significantly over calendar year for both sexes (smoothing terms overall *p*<0.001) in all groups except men winners, where variations were small. The greater smoothing terms were observed in top 101:200 group. In all finishers the trend was overall increasing, after decreasing in the first ten years. In S1 Fig, we showed, by gender and selection groups, the temporal trend line and the observed mean performances (plotted points) by calendar year. In Fig 1, multi-variable effects of temperature and calendar year on all finishers’ performance were shown by sex through perspective plot views. Therefore, one could observe the annual trend marathon by sex and the slowing-down of performance when average temperature increased. Analogously, in Fig 2, the multi-variable effects of wet-bulb globe temperature and year on all finishers’ performance were plotted by sex. In this case, as shown in Table 2, as wet-bulb globe temperature increased, performance worsened. In Fig 3, the bivariate effects of wind speed and pressure were shown for all finishers, near elite groups and winners. In Fig 4, the partial effects of temporal trend, keeping the other predictors constant, were plotted by wind direction for all finishers, near elite groups and winners. Curves were parallel, because in our model specification no interactions between year and weather conditions were considered. In Fig 5, the summed effects of country on race time were plotted by sex for all finishers and winners.

## Discussion

The aim of the present study was to investigate the role of weather conditions, together with sex and country, on performance in the Boston Marathon from 1972 to 2018. Different groups and subgroups of performance level were considered such as all runners, near elite 101:200 finishers, near elite 21:100, annual top ten finishers and annual winners. The main findings were (*i*) an increase of average temperature by 1 °C was related to worsened performance with greater effect for all finishers, (*ii*) increasing barometric pressure was related to worsened performances, (*iii*) wet-bulb globe temperature, analogously, when increasing was related to worsened performances, (*iv*) tail wind was related to improved performances of all groups, (*v*) precipitations, when increasing, were related to worsened performances, (*vi*) increasing wind speed was also related to worsened performances for all finishers and near elite groups.

### Increase of average temperature was related to worsened performance

A first important finding was that increasing temperature was related to impaired performance in almost all groups of runners (*i*.*e*. all finishers, top 101:200, top 21:100, and annual winners). It was well-known that there was a progressive slowing of marathon performance as the ambient temperature increased from 5 °C to 25 °C [3]. For this specific marathon, however, it has been observed that record breaking performances were achieved at a wet-bulb globe temperature of less than 7.8 °C, and 100% sky cover [7]. In contrast to these existing findings for Boston Marathon, a rather linear relationship was shown, where an increase of average temperature by 1 °C was related to worsened performance. Therefore, ambient temperature affected both elite and recreational runners in the same manner and there seemed to be a linear relationship between increase in ambient temperature and impairment in running performance.

### Increasing barometric pressure was related to worsened performances

A second important finding was that increasing barometric pressure worsened running performance. Most likely the increased barometric pressure is linked to an increased ambient temperature which is known to impair marathon running performance. Warm air causes air pressure to rise (https://sciencing.com/temperature-affect-barometric-pressure-5013070.html).

However, when the results of six European (*i*.*e*. Paris, London, Berlin) and American (*i*.*e*. Boston, Chicago, New York) marathon races from 2001 to 2010 through with 1,791,972 participants’ performances (all finishers per year and race) were analyzed, atmospheric pressure at sea level showed no effect on running performance [1]. Based on these disparate findings, future studies need to investigate more deeply the relationships between barometric pressure, air temperature and marathon running performance.

### Increasing wet-bulb globe temperature was related to worsened performances

A third important finding was that an increase in wet-bulb globe temperature was related to a worsened performance in all groups except annual winners.Wet-bulb globe temperature is nowadays the most widely used index of heat stress [16] in direct sunlight, which takes into account: temperature, humidity, wind speed, sun angle and cloud cover (solar radiation). This differs from the heat index, which takes into consideration temperature and humidity (www.weather.gov/tsa/wbgt). Little is known for the effect of wet-bulb globe temperature on marathon running performance. Ely et al. [6] reported a progressive slowing of marathon performance as the wet-bulb globe temperature increases from 5 °C to 25 °C for men and women of wide ranging abilities, but performance is more negatively affected for slower populations of runners. This was found when data from seven marathons (*i*.*e*. Boston, New York, Twin Cities, Grandma’s, Richmond, Hartford, and Vancouver Marathons) for a different range of years (*i*.*e*. 6 to 36 years) and different quartiles based on wet-bulb globe temperature and female and male finishers of different performance levels were analysed. The most likely explanation for the different findings is the fact that we analyzed a point-to-point race where wind might have a different effect on wet-bulb globe temperature compared to race held in one or several laps. Based on these disparate findings, future studies need to investigate more deeply the relationships between wet-bulb globe temperature and marathon running performance in other marathon races held in one or more laps.

### Tail wind was related to improved performances of all groups

A further important finding was that tail wind (*i*.*e*. wind from W, WNW, and WSW) improved race performance of all groups and increasing wind speed was also related to worsened performances for all finishers and near elite groups. Boston is a ‘point-to-point’ marathon from west to east (www.baa.org/races/boston-marathon/course-map). When the runners have a tailwind, they get a ‘push’ for 42 km. It is well-known for this race that headwinds on the day of the race slow winning times [4]. However, the important finding was that all performance groups seemed to profit from tailwind, not only the elite runners. Most likely only the Boston marathoners’ profit from wind direction, as other locations such as in Stockholm, showed there was no impact of wind on running performance [5]. A new aspect was that increased wind speed worsened performance for all finishers, which was not reported for finishers in the annual Stockholm Marathon from 1980 to 2008 [5].

### Variable role of precipitations

Another important finding was that increasing precipitations was related to worsened performances, but for annual winners it was not significant. In this specific race, a light drizzle was also conducive to better performances for record breaking performances [7]. In the Stockholm Marathon, the occurrence of rain was significantly and negatively related to finishing time anomaly, but the effects of rain only arose from the negative correlation with air temperature [5]. Based on these disparate findings, future studies would need to investigate more deeply the influence of precipitations on marathon running performance in runners of different performance levels.

### Sex and country differences

Further findings were that Kenyans and Ethiopians were the fastest nationalities and sex differences, with men faster in all groups, were greater in near elite groups. Country and sex effects, compared to the weather conditions effects, seemed to be more subjected to the selection criteria as their estimates varied between the performance groups more than the weather conditions estimates. For instance, sex differences varied from 00:17:25 h:min:sec (winners) to 00:41:51 h:min:sec (top 101:200 finishers). Differences between countries and sexes in all finishers, compared to the other selection groups, were relatively smaller. For instance, the interaction term USA:M reduced the comparison Kenya-Ethiopia and USA, for men, by 00:11:59 h:min:sec. This term was almost twice the respective term in annual winners group (00:06:07 h:min:sec). But in all finishers, the performance difference Kenya-Ethiopia and USA was of 01:36:43 h:min:sec, compared to 00:06:30 h:min:sec in the winners group. Therefore, the effect in the winners was greater.

### Limitations and strength

The findings of the present study were limited by the specific characteristics of this race in terms of environmental conditions (*e*.*g*. topography, temperature) and participation (*e*.*g*. qualifying criteria). Thus, they should be generalized with caution to other marathon races. Future studies would be needed to confirm these findings in other large city marathons. Moreover, no information about age was available and repeated measurements within runners could not be exactly identified, although it could be reasonably supposed that two observations belonged to the same runner if they had in common both name, surname, sex, country and participation, once a year, in the same period of time.

It should be also mentioned that Boston Marathon was the only large city marathon in the World with ‘qualifying times’ (www.baa.org/races/boston-marathon/participant-information/qualifying/history-of-qualifying-standards.aspx) and the qualifying standards could favour one sex and/or age group over the other. It was generally regarded that it was easier for women to qualify for Boston Marathon than men, which might influence the results. Although qualification criteria exist, about 10,000 runners participated annually on sponsor loyalties. Most likely, the number of this latter group of runners has increased most over the year, whilst qualifiers were stable over the years. It could be expected that the qualified age-groupers would perform better than their free-entry counterparts; however, with this information not available this speculative hypothesis could not be tested critically.

A further smaller point was the fact that for most years, women ran with men, thus they had pacers all along the course. For about the last 10 years, women have run alone, ahead of the men, i.e. they do not have pacers. This might have a small overall effect on relative male/female differences across the decades. Moreover, it was acknowledged that a logistic or limiting exponential model could also be used to examine trends over time [17–19]; however, the spline fit was applied since it was considered appropriate for all performance groups. On the other hand, strength of the study was that it analysed one of the most popular marathon races worldwide considering all finishers since the first women officially competed in this race. Since marathon running continues increasing its popularity, the findings would be of great interest for coaches and trainers working with marathon runners as well as for scientists focusing on human performance.

## Conclusions

For both female and male runners competing in Boston marathon between 1972 and 2018, an increase of average temperature by 1 °C and increasing barometric pressure were related to worsened performances with greater effect for all finishers, increasing wet-bulb globe temperature was related to worsened performances of all groups except winners, tail wind was related to improved performances of all groups, increasing wind speed was also related to worsened performances, but not for elite groups, and increasing precipitations worsened performances. Considering the selected groups of runners, weather conditions effects had less impact than country of origin and sex. Country and sex effects, compared to the weather conditions effects, seemed to be more subjected to the selection criteria as their estimates varied between the performance groups more than the weather conditions estimates. Future studies need to confirm these findings in other large city marathons such as the New York City Marathon.

## Supporting information

S1 Dataset Weather conditions in the Boston Marathon from 1972 to 2018. (XLSX)

S1 File R script to replicate the statistical analysis. (R)

S1 Table Frequency distribution of time-performance groups by sex and weather conditions (all finishers). (DOCX)

S1 Fig Performance, by sex and calendar year, of all finishers, near elite 101:200, near elite 21:100, top ten, winners. Points were observed average of time race. Lines were fitted curves.
(TIF)

## References

[1] El Helou N, Tafflet M, Berthelot G, Tolaini J, Marc A, Guillaume M, et al (2012) Impact of environmental parameters on Marathon running performance. PLoS ONE 7. 10.1371/journal.pone.0037407 PMC3359364 22649525

[2] Ely MR, Martin DE, Cheuvront SN, Montain SJ (2008) Effect of ambient temperature on marathon pacing is dependent on runner ability. Medicine and Science in Sports and Exercise 40: 1675–1680. 10.1249/MSS.0b013e3181788da9 18685522

[3] Ely MR, Cheuvront SN, Roberts WO, Montain SJ (2007) Impact of weather on marathon-running performance. Medicine and Science in Sports and Exercise 39: 487–493. 10.1249/mss.0b013e31802d3aba 17473775

[4] Miller-Rushing AJ, Primack RB, Phillips N, Kaufmann RK (2012) Effects of Warming Temperatures on Winning Times in the Boston Marathon. PLoS ONE 7. 10.1371/journal.pone.0043579 PMC3458848 23049738

[5] Vihma T (2010) Effects of weather on the performance of marathon runners. International Journal of Biometeorology 54: 297–306. 10.1007/s00484-009-0280-x 19937453

[6] Ely MR, Cheuvront SN, Montain SJ (2007) Neither cloud cover nor low solar loads are associated with fast marathon performance. Medicine and Science in Sports and Exercise 39: 2029–2035. 10.1249/mss.0b013e318149f2c3 17986912

[7] Trapasso LM, Cooper JD (1989) Record performances at the Boston Marathon: Biometeorological factors. International Journal of Biometeorology 33: 233–237. 2613367 10.1007/BF01051083

[8] Maughan RJ, Watson P, Shirreffs SM (2007) Heat and cold: What does the environment do to the marathon runner? Sports Medicine 37: 396–399. 10.2165/00007256-200737040-00032 17465618

[9] Montain SJ, Ely MR, Cheuvront SN (2007) Marathon performance in thermally stressing conditions. Sports Medicine 37: 320–323. 10.2165/00007256-200737040-00012 17465598

[10] Cheuvront SN, Haymes EM (2001) Thermoregulation and marathon running biological and environmental influences. Sports Medicine 31: 743–762. 10.2165/00007256-200131100-00004 11547895

[11] Maffetone PB, Malcata R, Rivera I, Laursen PB (2017) The Boston Marathon versus the World Marathon Majors. PLoS ONE 12. 10.1371/journal.pone.0184024 PMC5581174 28863152

[12] Knechtle B, Di Gangi S, Rüst CA, Nikolaidis PT (2018) The performance differences between the sexes in the Boston Marathon from 1972 to 2017. Journal of Strength and Conditioning Research. 10.1519/JSC.0000000000002760 30664107

[13] Knechtle B, Di Gangi S, Rust CA, Rosemann T, Nikolaidis PT (2018) Men’s Participation and Performance in the Boston Marathon from 1897 to 2017. Int J Sports Med 39: 1018–1027. 10.1055/a-0660-0061 30290371

[14] Cheuvront SN, Caruso EM, Heavens KR, Karis AJ, Santee WR, Troyanos C, et al (2015) Effect of WBGT Index Measurement Location on Heat Stress Category Classification. Med Sci Sports Exerc 47: 1958–1964. 10.1249/MSS.0000000000000624 25628176

[15] Angus SD, Waterhouse BJ (2011) Pacing strategy from high-frequency field data: more evidence for neural regulation? Med Sci Sports Exerc 43: 2405–2411. 10.1249/MSS.0b013e3182245367 21606868

[16] Budd GM (2008) Wet-bulb globe temperature (WBGT)—its history and its limitations. J Sci Med Sport 11: 20–32. 10.1016/j.jsams.2007.07.003 17765661

[17] Denny MW (2008) Limits to running speed in dogs, horses and humans. J Exp Biol 211: 3836–3849. 10.1242/jeb.024968 19043056

[18] Nevill AM, Whyte G (2005) Are there limits to running world records? Med Sci Sports Exerc 37: 1785–1788. 16260981 10.1249/01.mss.0000181676.62054.79

[19] Weiss M, Newman A, Whitmore C, Weiss S (2016) One hundred and fifty years of sprint and distance running—Past trends and future prospects. Eur J Sport Sci 16: 393–401. 10.1080/17461391.2015.1042526 26088705 PMC4867877

