# Supplementary material for: The role of weather conditions on running performance in the Boston Marathon from 1972 to 2018
Source: PLoS One. 2019 Mar 8;14(3):e0212797. doi: 10.1371/journal.pone.0212797 (PMC6407773; doi:10.1371/journal.pone.0212797)
Supplement: S1 Table — (DOCX) [file pone.0212797.s003.docx]

**S1 Table. Frequency distribution of time-performance groups by sex and weather conditions (all finishers)**

| \| **Women** \| \| \| \| \| \| \| \| \| \| **Men** \| \| \| \| \| \| \| \| \| \| --- \| --- \| --- \| --- \| --- \| --- \| --- \| --- \| --- \| --- \| --- \| --- \| --- \| --- \| --- \| --- \| --- \| --- \| --- \| \|  \| **(02:00,**  **02:30]** \| **(02:30, 03:00]** \| **(03:00, 03:30]** \| **(03:30, 04:00]** \| **(04:00, 04:30]** \| **(04:30, 05:00]** \| **(05:00, 05:30]** \| **(05:30, 06:00]** \| **> 06:00** \| **(02:00, 02:30]** \| **(02:30, 03:00]** \| **(03:00, 03:30]** \| **(03:30, 04:00]** \| **(04:00, 04:30]** \| **(04:30, 05:00]** \| **(05:00, 05:30]** \| **(05:30, 06:00]** \| **> 06:00** \| \| **N** \| 172 \| 2,038 \| 23,939 \| 75,984 \| 50,901 \| 24,744 \| 12,460 \| 6,796 \| 3,021 \| 3,682 \| 53,743 \| 126,158 \| 98,549 \| 47,904 \| 27,513 \| 13,488 \| 7,017 \| 2,879 \| \| **Temperature (C°)** \| N  (%) \| \| \| \| \| \| \| \| \| N  (%) \| \| \| \| \| \| \| \| \| \| 0-7 \| 28 (16.3) \| 427 (21.0) \| 5,219 (21.8) \| 15,485 (20.4) \| 10,623 (20.9) \| 5,481 (22.2) \| 3,173 (25.5) \| 1,744 (25.7) \| 1,101 (36.4) \| 738 (20.0) \| 12,106 (22.5) \| 26,616 (21.1) \| 20,460 (20.8) \| 11,021 (23.0) \| 7,112 (25.8) \| 3,931 (29.1) \| 2,095 (29.9) \| 1,154 (40.1) \| \| 8-15 \| 129 (75.0) \| 1,315 (64.5) \| 15,972 (66.7) \| 50,943 (67.0) \| 29,235 (57.4) \| 12,688 (51.3) \| 5,803 (46.6) \| 3,000 (44.1) \| 1,051 (34.8) \| 2,392 (65.0) \| 33,542 (62.4) \| 81,244 (64.4) \| 60,887 (61.8) \| 26,372 (55.1) \| 13,591 (49.4) \| 5,851 (43.4) \| 2,746 (39.1) \| 878 (30.5) \| \| 16-24 \| 15  (8.7) \| 296 (14.5) \| 2,748 (11.5) \| 9,556 (12.6) \| 11,043 (21.7) \| 6,575 (26.6) \| 3,484 (28.0) \| 2,052 (30.2) \| 869 (28.8) \| 552 (15.0) \| 8,095 (15.1) \| 18,298 (14.5) \| 17,202 (17.5) \| 10,511 (21.9) \| 6,810 (24.8) \| 3,706 (27.5) \| 2,176 (31.0) \| 847 (29.4) \| \| **Wind direction** \| N  (%) \| \| \| \| \| \| \| \| \| N  (%) \| \| \| \| \| \| \| \| \| \| Head wind \| 33 (19.2) \| 361 (17.7) \| 3,609 (15.1) \| 10,431 (13.7) \| 6,599 (13.0) \| 2,932 (11.8) \| 1,316 (10.6) \| 725 (10.7) \| 150 (5.0) \| 773 (21.0) \| 9,923 (18.5) \| 19,538 (15.5) \| 12,837 (13.0) \| 5,542 (11.6) \| 2,818 (10.2) \| 1,162 (8.6) \| 573 (8.2) \| 117 (4.1) \| \| Side wind \| 70 (40.7) \| 849 (41.7) \| 12,914 (53.9) \| 44,023 (57.9) \| 27,669 (54.4) \| 12,555 (50.7) \| 6,413 (51.5) \| 3,276 (48.2) \| 1,405 (46.5) \| 1,072 (29.1) \| 21,077 (39.2) \| 61,734 (48.9) \| 52,457 (53.2) \| 25,813 (53.9) \| 14,649 (53.2) \| 7,181 (53.2) \| 3,500 (49.9) \| 1,419 (49.3) \| \| Tail wind \| 69 (40.1) \| 828 (40.6) \| 7,416 (31.0) \| 21,530 (28.3) \| 16,633 (32.7) \| 9,257 (37.4) \| 4,731 (38.0) \| 2,795 (41.1) \| 1,466 (48.5) \| 1,837 (49.9) \| 22,743 (42.3) \| 44,886 (35.6) \| 33,255 (33.7) \| 16,549 (34.5) \| 10,046 (36.5) \| 5,145 (38.1) \| 2,944 (42.0) \| 1,343 (46.6) \| \| **Precipitations > 0 mm** \| 3  (1.7) \| 161 (7.9) \| 1,958 (8.2) \| 4,170 (5.5) \| 2,458 (4.8) \| 1,453 (5.9) \| 1,081 (8.7) \| 696 (10.2) \| 683 (22.6) \| 289 (7.8) \| 4,551 (8.5) \| 7,597 (6.0) \| 4,585 (4.7) \| 1,784 (3.7) \| 1,268 (4.6) \| 928 (6.9) \| 628 (8.9) \| 524 (18.2) \| \| **WBGT (%)** \| N  (%) \| \| \| \| \| \| \| \| \| N  (%) \| \| \| \| \| \| \| \| \| \| 0-6 \| 76 (44.2) \| 943 (46.3) \| 11,384 (47.6) \| 34,435 (45.3) \| 19,605 (38.5) \| 8,627 (34.9) \| 4,334 (34.8) \| 2,280 (33.5) \| 1,159 (38.4) \| 1,888 (51.3) \| 26,035 (48.4) \| 57,206 (45.3) \| 41,405 (42.0) \| 19,044 (39.8) \| 10,738 (39.0) \| 5,124 (38.0) \| 2,571 (36.6) \| 1,217 (42.3) \| \| 7-10 \| 70 (40.7) \| 803 (39.4) \| 9,588 (40.1) \| 30,731 (40.4) \| 20,648 (40.6) \| 10,027 (40.5) \| 5,059 (40.6) \| 2,698 (39.7) \| 995 (32.9) \| 1,314 (35.7) \| 20,725 (38.6) \| 50,205 (39.8) \| 38,712 (39.3) \| 18,425 (38.5) \| 10,310 (37.5) \| 5,116 (37.9) \| 2,596 (37.0) \| 817 (28.4) \| \| 11-15 \| 23 (13.4) \| 249 (12.2) \| 2,581 (10.8) \| 8,169 (10.8) \| 5,750 (11.3) \| 2,603 (10.5) \| 1,212 (9.7) \| 651 (9.6) \| 123 (4.1) \| 440 (12.0) \| 6,259 (11.6) \| 15,482 (12.3) \| 12,350 (12.5) \| 5,253 (11.0) \| 2,841 (10.3) \| 1,125 (8.3) \| 530 (7.6) \| 89  (3.1) \| \| 16-20 \| 3  (1.7) \| 43  (2.1) \| 386 (1.6) \| 2,649 (3.5) \| 4,898 (9.6) \| 3,487 (14.1) \| 1,855 (14.9) \| 1,167 (17.2) \| 744 (24.6) \| 40  (1.1) \| 724 (1.3) \| 3,265 (2.6) \| 6,082 (6.2) \| 5,182 (10.8) \| 3,624 (13.2) \| 2,123 (15.7) \| 1,320 (18.8) \| 756 (26.3) \| \| **Wind speed (km/h)** \| N  (%) \| \| \| \| \| \| \| \| \| N  (%) \| \| \| \| \| \| \| \| \| \| 9-17 \| 49 (28.5) \| 469 (23.0) \| 5,798 (24.2) \| 20,105 (26.5) \| 14,147 (27.8) \| 7,699 (31.1) \| 3,829 (30.7) \| 2,077 (30.6) \| 1,284 (42.5) \| 814 (22.1) \| 12,371 (23.0) \| 30,254 (24.0) \| 24,283 (24.6) \| 12,937 (27.0) \| 7,956 (28.9) \| 4,011 (29.7) \| 2,002 (28.5) \| 1,139 (39.6) \| \| 18-24 \| 73 (42.4) \| 1,055 (51.8) \| 10,388 (43.4) \| 29,979 (39.5) \| 19,394 (38.1) \| 8,296 (33.5) \| 4,083 (32.8) \| 2,082 (30.6) \| 602 (19.9) \| 2,229 (60.5) \| 30,713 (57.1) \| 63,022 (50.0) \| 45,417 (46.1) \| 20,144 (42.1) \| 10,841 (39.4) \| 5,127 (38.0) \| 2,572 (36.7) \| 766 (26.6) \| \| 25-39 \| 50 (29.1) \| 514 (25.2) \| 7,753 (32.4) \| 25,900 (34.1) \| 17,360 (34.1) \| 8,749 (35.4) \| 4,548 (36.5) \| 2,637 (38.8) \| 1,135 (37.6) \| 639 (17.4) \| 10,659 (19.8) \| 32,882 (26.1) \| 28,849 (29.3) \| 14,823 (30.9) \| 8,716 (31.7) \| 4,350 (32.3) \| 2,443 (34.8) \| 974 (33.8) \| \| **Pressure (hPa) ≥1015** \| 75 (45.7) \| 1,117 (37.2) \| 13,872 (46.8) \| 39,210 (50.6) \| 23,927 (49.9) \| 11,375 (50.2) \| 5,711 (51.9) \| 3,200 (54.4) \| 1,139 (59.8) \| 1,536 (41.0) \| 27,853 (50.0) \| 66,205 (51.8) \| 49,951 (49.8) \| 23,391 (50.1) \| 13,456 (52.1) \| 6,578 (54.5) \| 3,385 (55.2) \| 1,201 (64.0) \| |
| --- | --- | --- | --- | --- | --- | --- | --- | --- | --- | --- | --- | --- | --- | --- | --- | --- | --- | --- | --- | --- | --- | --- | --- | --- | --- | --- | --- | --- | --- | --- | --- | --- | --- | --- | --- | --- | --- | --- | --- | --- | --- | --- | --- | --- | --- | --- | --- | --- | --- | --- | --- | --- | --- | --- | --- | --- | --- | --- | --- | --- | --- | --- | --- | --- | --- | --- | --- | --- | --- | --- | --- | --- | --- | --- | --- | --- | --- | --- | --- | --- | --- | --- | --- | --- | --- | --- | --- | --- | --- | --- | --- | --- | --- | --- | --- | --- | --- | --- | --- | --- | --- | --- | --- | --- | --- | --- | --- | --- | --- | --- | --- | --- | --- | --- | --- | --- | --- | --- | --- | --- | --- | --- | --- | --- | --- | --- | --- | --- | --- | --- | --- | --- | --- | --- | --- | --- | --- | --- | --- | --- | --- | --- | --- | --- | --- | --- | --- | --- | --- | --- | --- | --- | --- | --- | --- | --- | --- | --- | --- | --- | --- | --- | --- | --- | --- | --- | --- | --- | --- | --- | --- | --- | --- | --- | --- | --- | --- | --- | --- | --- | --- | --- | --- | --- | --- | --- | --- | --- | --- | --- | --- | --- | --- | --- | --- | --- | --- | --- | --- | --- | --- | --- | --- | --- | --- | --- | --- | --- | --- | --- | --- | --- | --- | --- | --- | --- | --- | --- | --- | --- | --- | --- | --- | --- | --- | --- | --- | --- | --- | --- | --- | --- | --- | --- | --- | --- | --- | --- | --- | --- | --- | --- | --- | --- | --- | --- | --- | --- | --- | --- | --- | --- | --- | --- | --- | --- | --- | --- | --- | --- | --- | --- | --- | --- | --- | --- | --- | --- | --- | --- | --- | --- | --- | --- | --- | --- | --- | --- | --- | --- | --- | --- | --- | --- | --- | --- | --- | --- | --- | --- | --- | --- | --- | --- | --- | --- | --- | --- | --- | --- | --- | --- | --- | --- | --- | --- | --- | --- | --- | --- | --- | --- | --- | --- | --- | --- | --- | --- | --- | --- | --- | --- | --- | --- | --- | --- | --- | --- | --- | --- | --- | --- | --- | --- | --- | --- | --- | --- | --- | --- | --- | --- | --- | --- | --- | --- | --- | --- | --- | --- | --- | --- | --- | --- | --- | --- | --- | --- | --- | --- | --- | --- | --- | --- | --- | --- | --- | --- | --- | --- | --- | --- | --- | --- | --- | --- | --- | --- | --- | --- | --- | --- | --- | --- | --- | --- | --- | --- | --- | --- | --- | --- | --- | --- | --- | --- | --- | --- | --- | --- | --- | --- | --- | --- | --- | --- | --- | --- | --- | --- | --- | --- | --- | --- | --- | --- | --- | --- |
