# Supplementary figures and images for: The role of weather conditions on running performance in the Boston Marathon from 1972 to 2018
Source: PLoS One. 2019 Mar 8;14(3):e0212797. doi: 10.1371/journal.pone.0212797 (PMC6407773; doi:10.1371/journal.pone.0212797)

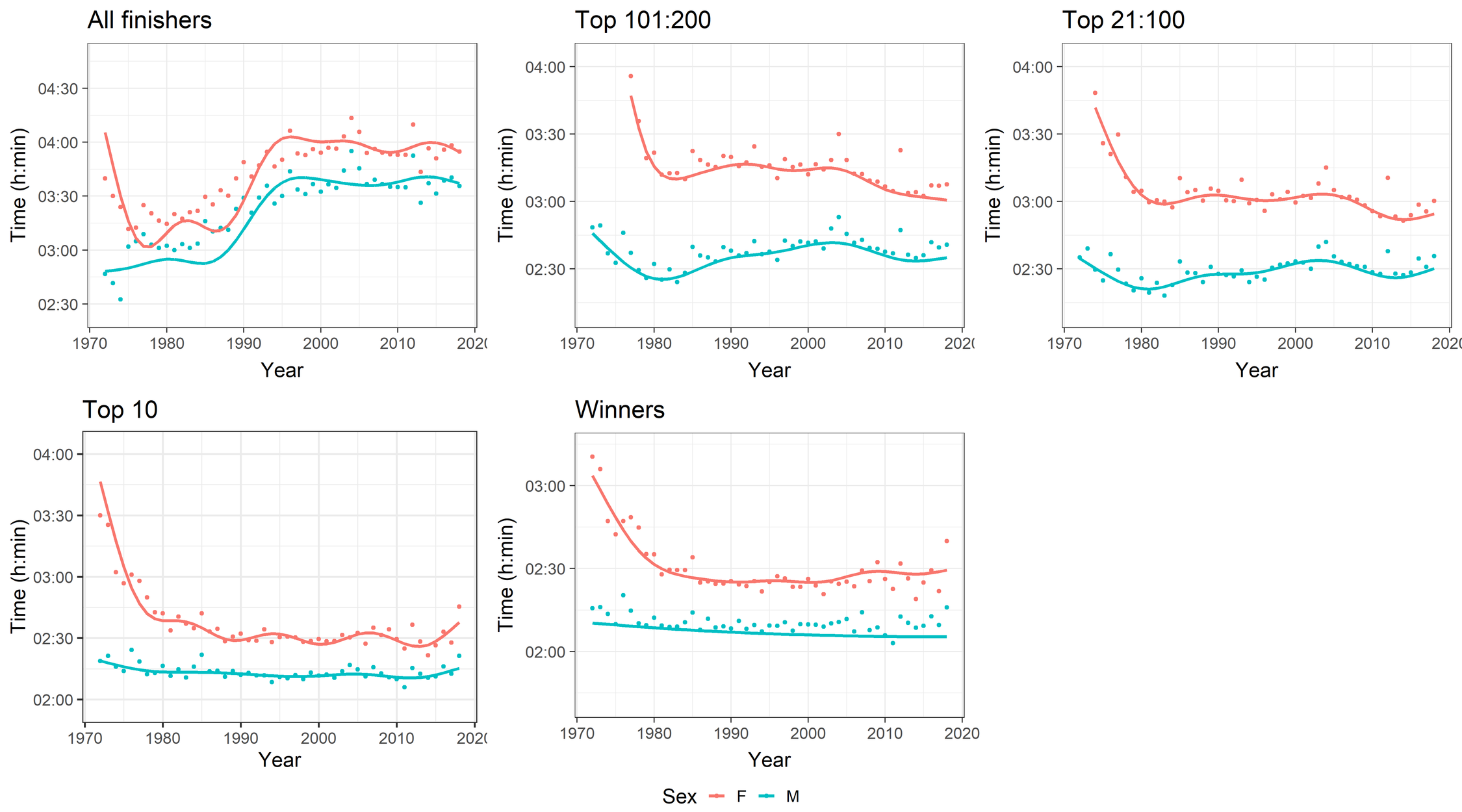

Supplement: S1 Fig — Points were observed average of time race. Lines were fitted curves. (TIF) [file pone.0212797.s004.tif]
